# Wireless intraoral tongue control of an assistive robotic arm for individuals with tetraplegia

**DOI:** 10.1186/s12984-017-0330-2

**Published:** 2017-11-06

**Authors:** Lotte N. S. Andreasen Struijk, Line Lindhardt Egsgaard, Romulus Lontis, Michael Gaihede, Bo Bentsen

**Affiliations:** 1 Center for Sensory Motor Interaction, Department of Health Science and Technology, Aalborg University, Aalborg, Denmark; 2 Department of Otolaryngology, Head and Neck Surgery, Aalborg University Hospital, Aalborg, Denmark; 3 Department of Clinical Medicine, Aalborg University, Aalborg, Denmark

**Keywords:** Tongue interface, Assistive robotic arm, Rehabilitation, Disabilities, Tetraplegia, Assistive devices

## Abstract

**Background:**

For an individual with tetraplegia assistive robotic arms provide a potentially invaluable opportunity for rehabilitation. However, there is a lack of available control methods to allow these individuals to fully control the assistive arms.

**Methods:**

Here we show that it is possible for an individual with tetraplegia to use the tongue to fully control all 14 movements of an assistive robotic arm in a three dimensional space using a wireless intraoral control system, thus allowing for numerous activities of daily living. We developed a tongue-based robotic control method incorporating a multi-sensor inductive tongue interface. One abled-bodied individual and one individual with tetraplegia performed a proof of concept study by controlling the robot with their tongue using direct actuator control and endpoint control, respectively.

**Results:**

After 30 min of training, the able-bodied experimental participant tongue controlled the assistive robot to pick up a roll of tape in 80% of the attempts. Further, the individual with tetraplegia succeeded in fully tongue controlling the assistive robot to reach for and touch a roll of tape in 100% of the attempts and to pick up the roll in 50% of the attempts. Furthermore, she controlled the robot to grasp a bottle of water and pour its contents into a cup; her first functional action in 19 years.

**Conclusion:**

To our knowledge, this is the first time that an individual with tetraplegia has been able to fully control an assistive robotic arm using a wireless intraoral tongue interface. The tongue interface used to control the robot is currently available for control of computers and of powered wheelchairs, and the robot employed in this study is also commercially available. Therefore, the presented results may translate into available solutions within reasonable time.

## Background

**Fig. 1:**
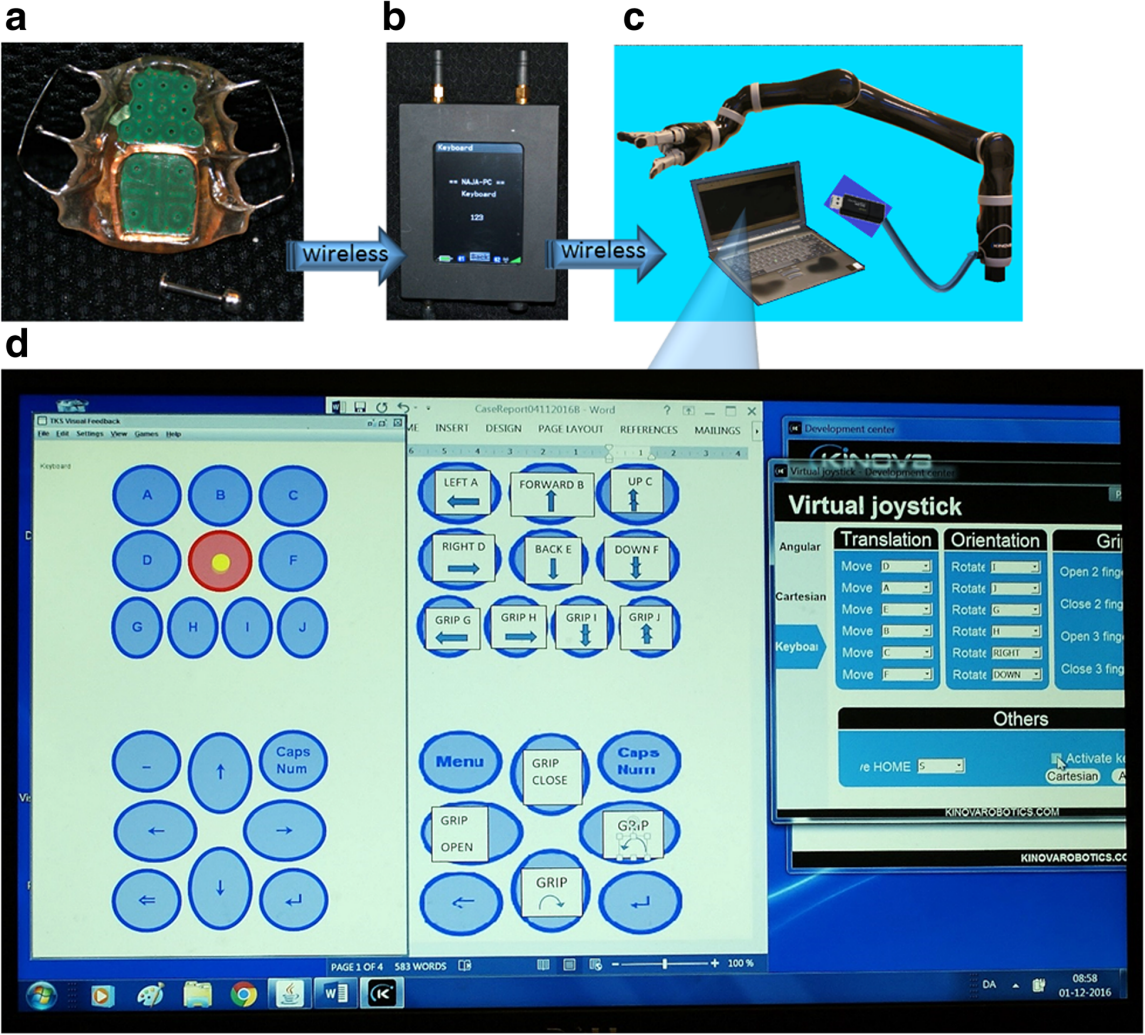
Overview of the tongue-based robotic control system. **a** The inductive tongue interface incorporating 10 sensors in the keypad and 8 sensors in the mousepad. The sensors are activated using a glued or pierced metal activation unit shown at the bottom. **b** The central unit which wirelessly receives the signal from the tongue interface and transforms these into characters. **c** The computer which wirelessly receives the characters from the central unit and transforms these into commands. The commands are passed on to the assistive robotic arm through the USB port. **d** The computer screen showing the visual feedback to the experimental participant (left), the mapping of the sensors to the movement of the robot (middle) and the robot software (right)

Advanced assistive robotic arms have been developed to support the daily living of disabled individuals, [1–6] but in case of tetraplegia, the control of these assistive arms is challenged as they are usually controlled by a joystick. At the same time, the ability to control assistive robotic arms may radically change the self-supportiveness of these severely paralyzed individuals and fundamentally increase their quality of life [1–3]. Some research has focused on semi-automated control of robotic arms using brain-machine interfaces with promising results in laboratory settings [7, 8]. However, partly due to their often invasive character and the challenging nature of the neural recordings, those efforts have not yet resulted in systems that can be used at home by individuals with tetraplegia. Others have demonstrated the use of extra oral interfaces to control limited movements of robotic arms in task specific set-ups [9, 10]. In the recent years, new intra oral tongue based computer interfaces have been proposed for control of computers [11–16]. The high flexibility of the tongue allows for the precise selection of intraoral sensors to generate highly reliable control signals. Further, the cranial nervous innervation of the tongue often leaves its sensory motor control intact after high level spinal cord injuries, which may elsewise leave a patient with completely paralyzed limbs. Still, none of the current systems have demonstrated the ability to provide sufficient control signals for full 3-dimensional control of an assistive robot with a functionality comparable to the human arm. This is important in order to make such assistive robotic arms fully functional in daily living. An example of an assistive robot which has a mobility similar to the human arm is the JACO assistive robotic arm with a gripper [4] (Fig. 1c). Such mobility demands 14 control signals. Thus, there is an urgent need for control systems allowing paralyzed individuals to voluntarily control the high number of robotic motions required to perform activities of daily living with an assistive robot.

Therefore, we developed a new assistive robotic control method for individuals with C-level spinal cord injury, who usually have preserved full sensory-motor control of their tongue.

## Methods

The tongue was used for volitional input to an assistive robotic arm in order to provide full control of the robots 12 possible movements and of its three-finger gripper.

### Study participants

One 37-year-old able-bodied female, E1, performed direct actuator control. This participant had previously used the tongue control system to control a computer [12, 17] and a wheelchair [18] on five experimental days more than 1 year prior to the current experiment. The previous experiments with the tongue control systems were performed with an activation unit pierced to the tongue, but in the current study the activation unit was glued to the tongue with tissue glue (Histoacryl®) since the participant no longer had a tongue piercing. A custom-made tongue interface (Fig. 1a) was produced from a cast of her upper mouth. The tongue interface was based on a removable dental appliance comparable with a removable partial denture or orthodontic appliance, and it was produced using standard dental materials [5].

One 64-year-old female, E2, with tetraplegia performed endpoint robotic control. Her tetraplegia was due to a spinal cord tumour at level C1-C2 resulting in SCI (spinal cord injury) 19 years prior to the current study. Her SCI was incomplete, but she was unable to move her extremities and trunk except from slight non-functional motions of her fingers.

This individual participated in earlier experiments and she was pierced 6 years ago at Aalborg University Hospital; due to comorbidity of these individuals, their piercings were performed by health professionals, ie. a dentist and an otolaryngology specialist, and the she was admitted for 24-h in the hospital for observation of swelling and bleeding of the tongue [13]. The tongue piercing provided a permanent solution for activation of the sensors, and this individual had her own tongue computer interface, which she mainly used for wheelchair control employing the mousepad of the tongue interface (Fig. 1a).

### Tongue-robot interfacing

The tongue-robot interface developed in this study was based on the inductive tongue control system, which has previously been developed for control of computers [11, 12, 17], powered wheelchairs [18, 19] and prosthetic devices [20]. A commercially available version of this system, iTongue [14] (Fig. 1a, b), was modified to provide continuous character input to a computer, which executed software (Fig. 1d) to transform these characters to command signals for an assistive robotic arm, JACO [3, 4], with a three-finger gripper (Fig. 1c). Two versions of tongue-based robotic control schemes were implemented: Direct actuator control in order to demonstrate the complete volitional control of the robot provided by the tongue-robot interface and further Cartesian endpoint control was implemented to demonstrate a clinical and more intuitive application of the system.

The inductive tongue control system consists of two main parts: a tongue interface resembling an intraoral dental appliance mounted at the hard palate (Fig. 1a) and an external central unit (Fig. 1b). The intraoral plate carried 18 inductive sensors together with the required electronic circuit to record signals from sensors and to transmit the sensor signals wirelessly out of the oral cavity. In addition, the tongue interface includes a rechargeable battery and a charger coil for wireless charging. The 18 inductive sensors are configured in a mousepad consisting of 8 sensors and a keypad consisting of 10 sensors. The keypad is designed for direct text typing on a computer with several characters relating to each sensor as in old mobile phones, see [12] for details. The mousepad is designed to control the cursor, while using a computer or to control a powered wheelchair. The signals of the eight sensors are interpolated by the external central unit to obtain a joystick-like functionality, when the computer cursor or the powered wheelchair is being controlled [12]. The central unit allows the user to choose which device to control; either a computer/tablet/smartphone or a powered wheelchair by changing between keypad mode, mousepad mode and wheelchair mode. If the keypad mode is chosen, the 8 sensors of the mousepad can be activated individually like the 10 sensors in the keypad, giving a total of 18 possible command signals for robotic control. If used for computer control, the central unit emulates a standard wireless keyboard and mouse through a Bluetooth connection to the computer.

In order to employ the inductive tongue-computer interface for robotic control several changes of the iTongue system were implemented:

Firstly, direct robotic control of the 7 degrees of freedoms of the robot with the three-finger gripper required the use of 14 out of the 18 sensors of the inductive tongue-computer interface as single buttons providing a continuous control signal as long as the sensors were activated. Therefore, a robotic keypad configuration was implemented, which employed sensors from both the keypad area and the mouse pad area of the tongue interface (Fig. 1d). Further, the iTongue system provides feedback to the user by displaying the activated character at the cursor for a period of time before it is actually typed in order to give the user time to deselect the sensor without typing if the desired character was not selected [12]. This is implemented by sending a character to the computer when a sensor is activated followed by a backspace, if the sensor is deselected within a certain time period. This feature was disabled in the current study in order to obtain a continuous flow of characters as long as a sensor was activated. Finally, a 0.9–1 s dwell time was introduced for the initial character input to the computer in order to avoid activation of the robot when the user was talking or swallowing which could briefly activate a sensor.

The central unit of the iTongue system was further modified to transmit all received sensor data to the COM port of the computer in addition to the emulated keyboard characters. This included amplitudes of all sensor signals and was implemented through a software program, which in addition provided feedback to the user showing which sensor was activated (Fig. 1d, left window).

**Fig. 2:**
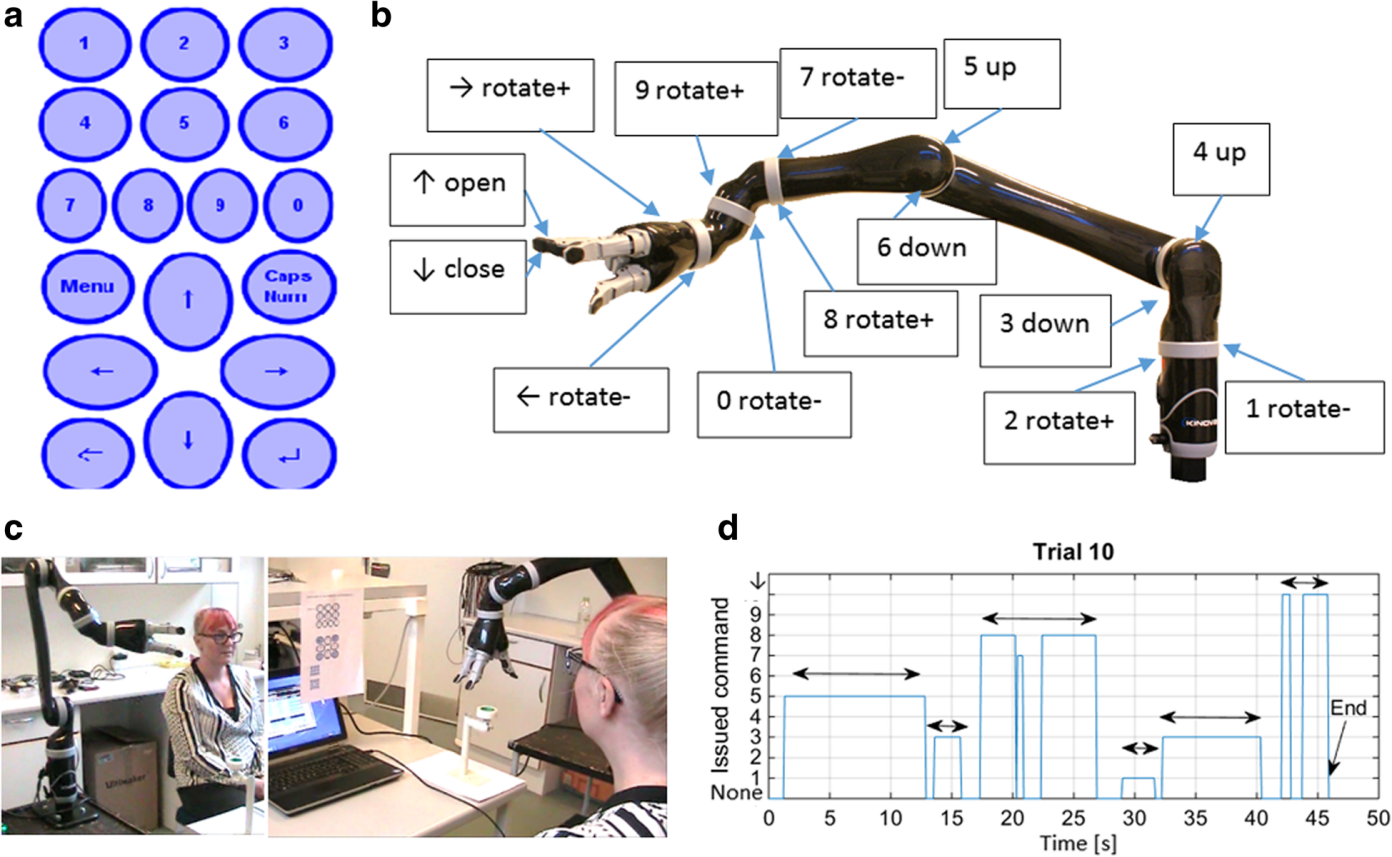
Experimental setup and results for direct tongue-robot actuator control. **a** Characters and functions assigned to the sensors of the tongue interface (**b**). The mapping of the sensors of the tongue interface to the robot movements (**c**). The assistive robotic arm in the “home position” next to E1 who is wearing the tongue interface (left) and the experimental set-up for the functional task of picking up a roll of tape placed on a metal holder (**d**). The sequence of issued commands during a functional task. The arrows indicate the intended duration of a command. The commands refer to the character-command map shown in (**b**)

The mapping of the sensors of the tongue interface to the movements of the robot was implemented using the Virtual joystick software from Kinova [4] (Fig. 1d, right window). Two types of mapping were implemented; direct actuator control and endpoint control. The direct actuator control was implemented as shown in Fig. 2a,b. This implementation was used to demonstrate that the user had full control of each of the seven actuators used in the robot. The mapping was performed based on knowledge from previous studies of the tongue control system showing that sensors at the front of the palatal area are easier to reach than sensors at the back of the palatal area [21, 22]. This control scheme was used by the abled-bodied experimental participant, E1.

The sensor-robot mapping for the endpoint control (Fig. 1d, middle window) was implemented using the Cartesian settings in the Virtual joystick software (Fig. 1d, right window). Again, the most frontal sensors were prioritized and in addition it was prioritized that two adjacent sensors were used to control opposite motions in order to make the control intuitive. At the pre-experiment with the participant with tetraplegia each sensor– actuator map was tested and adjusted according to the personal preferences, in order to further increase the intuitiveness of the robotic control.

### Experimental procedure and data acquisition

**Fig. 3:**
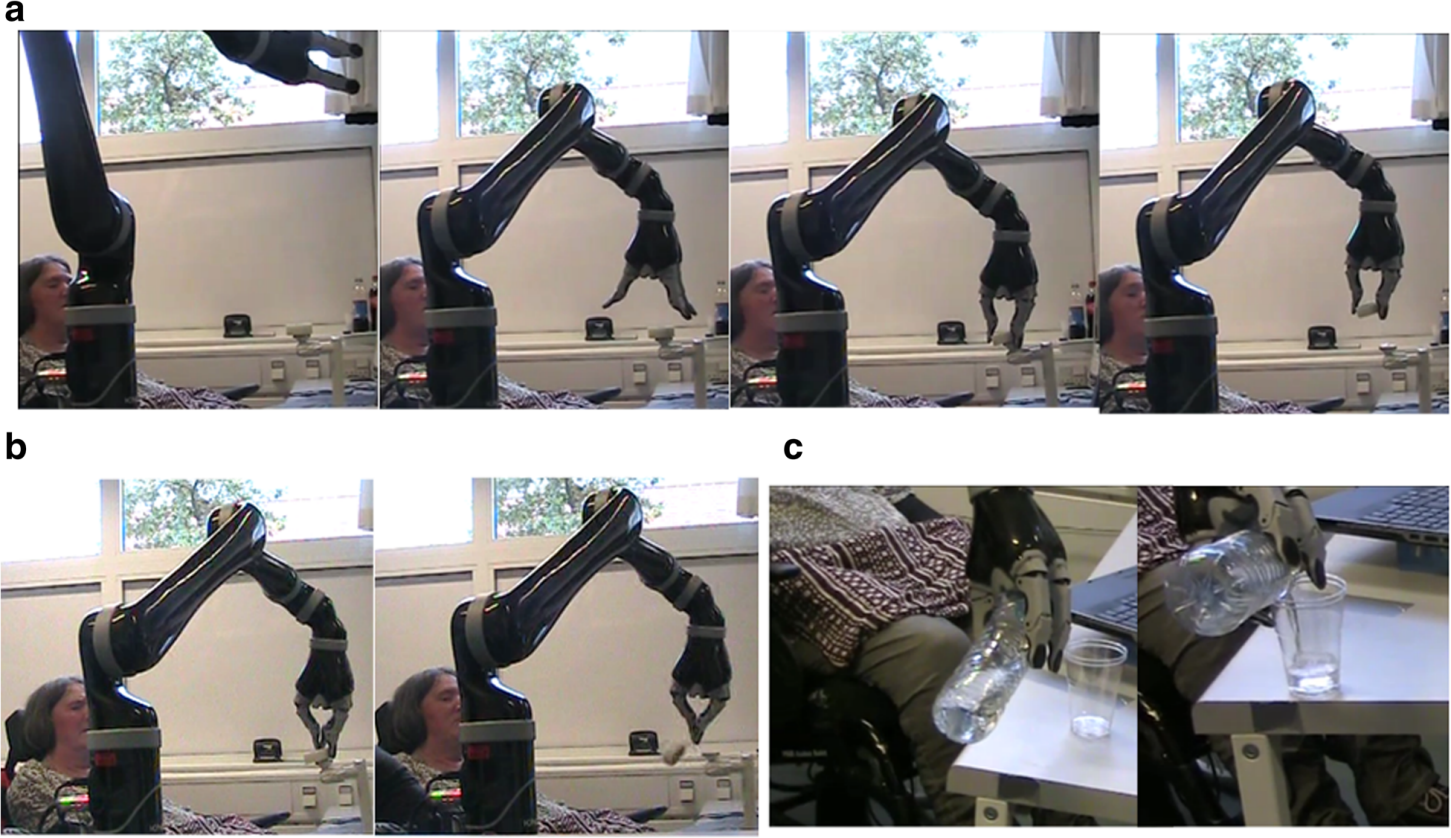
Tongue based endpoint control of the assistive robotic arm by E2. **a** Successful reach and grasp of the roll of tape by E2 sitting in her wheelchair to the left of in the picture (**b**). Successful reach and touch of the roll of tape, but the roll was dropped during lift off (**c**). E2 poring a cup of water for the first time in 19 years

During the experiments the participants each wore their custom-made tongue interface and sat next to the robot, which was placed on a mobile table (Figs. 2c, and 3). A computer running the visual feedback to the user, displaying the sensor-robot map, and running the Virtual joystick software was placed on a table in front of the participant (Fig. 1d). Further, an enlarged image of the sensor-robot map was placed in front of the participant. A roll of tape (6 cm diameter) was placed on the top of a holder, which allowed the roll of tape to slide off, if it was pushed by the assistive robot (Fig. 2c).

First, we demonstrated the ability of the inductive tongue control system to control the 14 possible movements of the assistive robotic arm and gripper by directly mapping the sensors of the tongue interface to the motors of the robotic arm (Fig. 2a,b).

Next, we conducted a proof of concept study exploiting the human ability to control all of the 14 possible movements of the assistive robotic arm with the tongue by letting E1 control functional movements of the assistive robot. For both E1 performing direct control, and E2 performing end-point control, the functional movements consisted of reaching for and grasping the roll of tape placed on the holder (Fig. 2c, right).

Finally, we tested the more intuitive control scheme with Cartesian control of the end-point of the assistive robotic arm (Fig. 1d). This more functional application of the tongue control of the assistive robotic arm was demonstrated clinically by E2 (Fig. 3). Since E2 brought her own tongue interface for the experiment, a pre-experimental day was conducted in order to set-up and test the mapping of the robot and the sensors of her personal tongue control system, and further to integrate her own tongue interface with an experimental central unit adapted to perform assistive robotic control (Fig. 1d). The sensor-robot mapping was tested by letting E2 activate the sensors of the tongue interface sequentially to confirm that the assisting robotic arm was moving accordingly. To make E2 comfortable with the assistive robotic arm, the endpoint velocity of the robotic arm was set to 0.07 m/s only whereas the velocity was 0.20 m/s for E1.

After the pre-experimental day, we proceeded the following day with the clinical proof-of-concept study which included a training period followed by 10 trials of performing the functional task. E2 was positioned out of reach of the robotic arm for safety reasons. Due to her position in her wheelchair, this resulted in a distance of more than 1.5 m between her eyes and the roll of tape to be picked up by the assistive robotic arm.

Both E1 and E2 were given a 30 min training period to train the robotic control prior to the performance of the functional tasks. This training period consisted of 4 steps. Step 1 was to activate all the possible commands in a sequential manner to ensure this was possible and to identify the most difficult activations. Step 2 was to practice the most difficult activations. Step 3 was to practice arbitrary commands as chosen by the participant in order to get familiar with the robotic movements. Step 4 was initiated when the participant felt confident with the robotic movements and included practice of the functional task. The training session was concluded after a total training time of 30 min.

At the beginning of each functional trial, the robot was positioned in a “home” position (Fig. 2c, left) which required the experimental participants to perform several movements with the robot in order to reach for and grasp the roll of tape.

### Data acquisition and processing

The trials were recorded with a video camera. Further, amplitude data from the sensors of the tongue interface were transmitted to the COM port of the computer 30 times per second and saved. The video files were analysed to determine task completion time, number of issued control signals, and type of issued control signals. If the same command was issued several times in a row, it was counted as one command; e.g. at the end of trial 10 of E1, the gripper was closed followed by a short break and then the gripper was closed further (Fig. 2d). This was counted as a single command to close the gripper to obtain the results shown in Table 1. In cases where the gripper of the robot was closed at the beginning of a trial, the opening command for the gripper was not counted. If the roll of tape was picked-up successfully, it was counted as “picked-up”. If the robot gripper touched the roll of tape, but did not manage to lift it from the holder without dropping it, it was counted as a “touch”. Mean values ± Standard Deviations were calculated for the number of issued control signals and for the trial completion time (Table 1).

**Table 1:** Results of tongue-controlled robotic grasping, number of trials = 10

Participant	Robot control method	Robot translational velocity [m/s]	Roll picked up [Times]	Roll touched but dropped [Times]	Completion time: Mean ± STD^b^	No. of issued commands^a^: Mean ± STD^b^
E1	Direct actuator	0.20	8	2	71.3 ± 16.7	17.6 ± 5.5
E2	Cartesian endpoint	0.07	5	5	70.1 ± 15.3	6.0 ± 1.5

^a^When the same command was issued several times in a row, it was only counted as one command. ^b^STD Standard deviation

## Results

### Tongue based robotic control by E1

After 30 min og training, the participant, E1, was able to activate and control all of the 14 robotic movements and to pick up the roll of tape in 80% of the 10 attempts by sequentially activating the motors of the robot by the use of her tongue (Fig. 2c, d). Mean (±SD) completion time and number of issued commands were 71.3 ± 16.7 s and 17.6 ± 5.5 s respectively (Table 1). An example of the sequence and duration of the issued commands is shown in Fig. 2d.

### Tongue based robotic control by E2

As the test of each single sensor-robot mapping was concluded on the pre experimental day, E2 realized that she could now fully control the assistive robotic arm, and she immediately proceeded to control the robot to perform a handshake with the experimenter. This was her first handshake since her spinal cord injury 19 years ago.

**Fig. 4:**
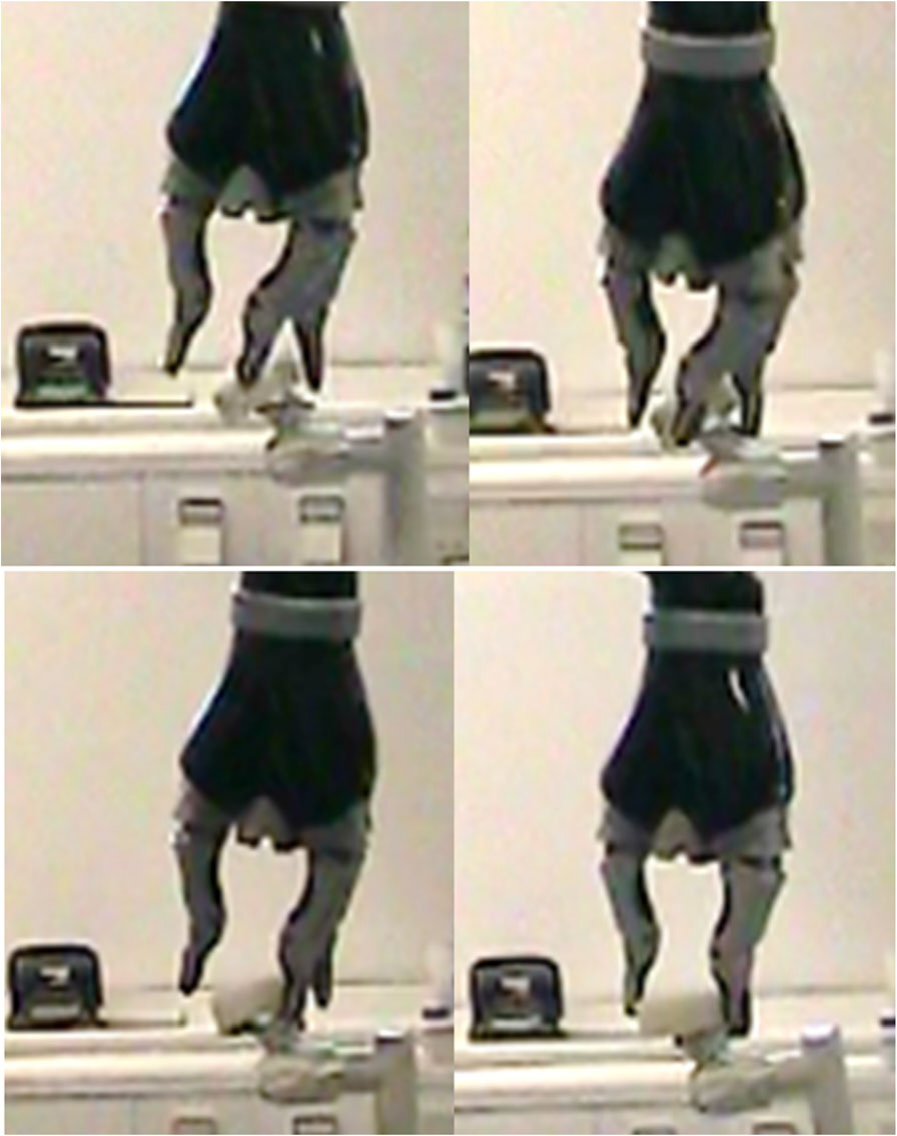
The unsuccessful pick-up of the roll of tape by E2, classified as touch. The 5th trial classified as touch is shown in Fig. 3b

After 30 min. of training on the experimental day, E2 was able to reach out and touch the roll of tape in 100% of the 10 attempts, and she also picked up the roll in 50% of these attempts. Mean (±SD) completion time and number of issued command were 70.1 ± 15.3 s and 6.0 ± 1.5 respectively (Table 1). In the cases where E2 did not successfully pick up the roll, she had difficulties seeing whether she had positioned the gripper correctly in order to grasp the roll of tape due to the distance between her eyes and the robot. An example of a successful attempt is shown in Fig. 3a. The five unsuccessful attempts, trials number 1,3,4,6 and 7, which were classified as “touch” are shown in Fig. 3b. (trial 6) and in Fig. 4.

At the end of the experiment, E2 decided to pick up a bottle of water with the assistive robotic arm and to pour its contents into a plastic cup. She was successful in performing this action in her first attempt (Fig. 3c). She expressed a strong desire to bring the assistive robot home. Among others, her personal desires were to use the robotic arm to take her own clothes out of the cabinet, eat and drink by herself, play social games with her family and to “hold hands” with her grandchildren. She claimed that the robot would allow her to open doors. Therefore, she would not mind spending time on her own, and thus reducing the need of a personal assistant.

**Fig. 5:**
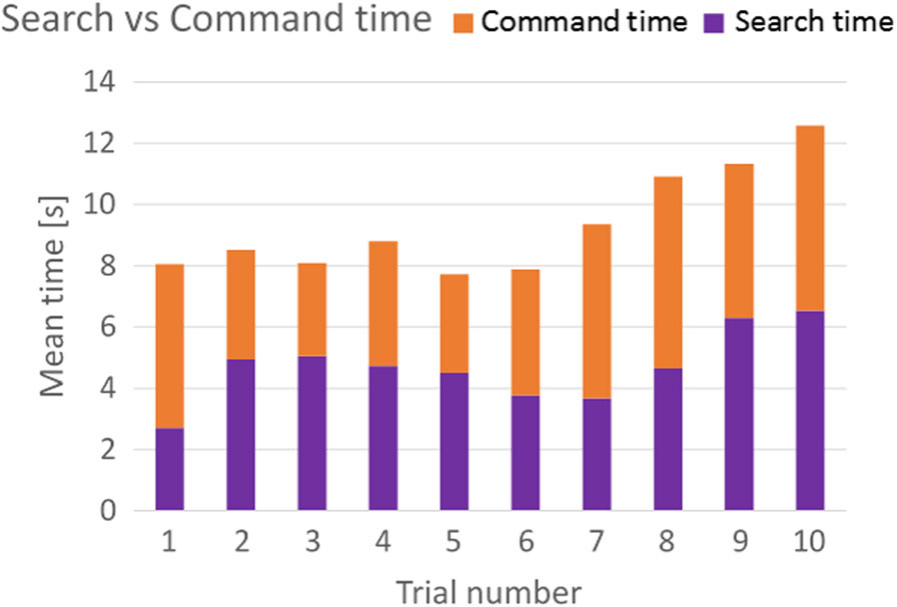
The average time spend between two commands is shown in together with the average duration of the resulting robotic movement for participant E2

The average time spent between two commands is shown in Fig. 5 together with the average duration of the resulting robotic movement. The time between the commands included the time to study the displayed map between tongue sensors and robotic movements in order to decide which sensor to activate, the time needed for location of the sensor by the tongue, and the 0.9 s dwell time before a sensor selection initiated a robotic motion.

## Discussion

To our knowledge, this study is the first to demonstrate that it is possible for an individual with tetraplegia to use the tongue intraorally to fully control all 14 motions of a robotic arm with a mobility comparable to the human arm. This facilitates the use of any task the robotic arm can perform including activities of daily living. During use, the tongue interface is practically invisible due to its intraoral location and thus aesthetically acceptable for user.

It is expected that further training and increase of robotic velocity will improve the completion time of the different tasks performed with the robot. We suggest that future implementations of the system facilitate a more joystick-like control by incorporating the mousepad of the tongue control system since such control may be more useful [23] and a combination of preprogramed movements of the assistive robotic arm and of voluntary control in order to assist finalizing voluntarily controlled movements.

Since both the robotic arm and a similar version of the inductive tongue control system as deployed in this study are currently available for individuals with tetraplegia, the results of this study may very well translate into an available solution within reasonable time.

## Conclusion

The presented method provides a solution in which a paralyzed individual can control a computer, [11, 12, 17] a wheelchair [18, 19] and an assistive robotic arm with the same tongue interface without the need of calibration, long-term training or the need of intervention from a helper. Thus, the presented system may possibly challenge the current need for 24 h personal assistance of individuals with tetraplegia and significantly improve their quality of life through their empowerment. Future studies should evaluate the suggested method in relation to activities of daily living and through more standardized assessment methods [23] including e.g. assessment of throughput [21, 24] and the path efficiency [25].

## Abbreviations

SCI: Spinal cord injury
STD: Standard deviation
